# Impact of Drugs Used in Intensive Care on Routine Coagulation Testing

**DOI:** 10.3390/diagnostics15070941

**Published:** 2025-04-07

**Authors:** Joffrey Feriel, Marjorie A. Goujon, Miki Desez, François Depasse

**Affiliations:** Clinical Development—Medical Affairs Department, Diagnostica Stago, 3 allée Thérésa, 92600 Asnieres sur Seine, France; marjorie.goujon.ext@stago.com (M.A.G.); miki.desez@stago.com (M.D.); francois.depasse@stago.com (F.D.)

**Keywords:** intensive care unit (ICU), drugs, interference, hemostasis, prothrombin time (PT), activated partial thromboplastin time (aPTT), anti-Xa, fibrinogen, chromogenic assays, chronometric assays

## Abstract

Coagulation testing is commonly used in the intensive care unit (ICU) to monitor and manage the hemostatic balance, assess bleeding risk, and guide anticoagulant therapy. Routine tests used for this purpose include prothrombin time, activated partial thromboplastin time, fibrinogen, and anti-Xa assays. Some of the drugs commonly used in critically ill patients may influence coagulation assays by interacting in vitro with reagents or in vivo with coagulation pathways, thus altering the coagulation cascade and the fibrinolytic pathway. While the pharmacological effects of drugs on coagulation are usually documented, to our knowledge, no comprehensive review article has been published to date. In this review, we have conducted a critical analysis of the literature to define: (1) the impact of hydroxocobalamin, intravenous lipid emulsion, and propofol on chromogenic assays; (2) the impact of PEGylated compounds, emicizumab, recombinant activated factor VII, antibiotics, and sugammadex on chronometric assays; (3) the challenges associated with bridging anticoagulation in the ICU as well as the effect of N-acetylcystein, serotonin reuptake inhibitors, and tramadol on the hemostasis system. For each drug, we specify the routine coagulation assay that is impacted, whether this is linked to an in vitro interference or an in vivo effect, and the potential consequences on patient management.

## 1. Introduction

Coagulation testing is commonly used in the intensive care unit (ICU) to monitor and manage the hemostatic balance, assess bleeding risk, and guide anticoagulant therapy, especially in critically ill patients who may present with severe coagulopathies. Routine coagulation tests, such as prothrombin time (PT), activated partial thromboplastin time (aPTT), fibrinogen, and anti-Xa assays, are routinely used in the ICU for this purpose [1]. Drugs administered in the ICU, especially anticoagulants but also many others commonly used in the ICU, such as thrombolytics, antibiotics, or antiplatelet drugs, can directly or indirectly alter coagulation parameters [2]. They may influence coagulation assays by interacting in vitro with reagents or in vivo with coagulation pathways, thus altering the coagulation cascade and the fibrinolytic pathway [3]. In addition, patients in the ICU may have underlying diseases such as liver or renal dysfunction, which may also directly or indirectly impact the hemostatic balance. Variability is also observed across laboratory methods that may make diagnosis even more challenging. While the pharmacological effects of drugs on coagulation are usually documented, the comprehensive impact of drugs used in the ICU has not been systematically reviewed. However, understanding these effects is critical for ensuring that coagulation tests accurately reflect the patient’s actual hemostatic status and are used effectively for clinical decision making. Emerging analytical techniques could help identify or reduce drug-induced test interference. An HIL measurement module, available on some coagulation analyzers, can identify optical interference and switch to an alternative wavelength for some of them. Interference with phospholipids often depends on their concentration or the presence of a specific subtype; changing the composition of certain reagents could reduce the impact of certain drugs and improve patient care. Pre-analytical, analytical, and post-analytical variables impacting D-dimer results are a broad topic that has been successively summarized elsewhere, so we chose to exclude them from our review [4].

In this narrative review, we will define which drugs used in intensive care can interfere with chromogenic and chronometric assays, as well as those affecting the hemostasis system and complicating the interpretation of routine coagulation testing. We aim to provide a comprehensive overview of these drugs by specifying which assays they impact, whether this is linked to in vitro interference or in vivo effects, and the potential consequences on patient management.

## 2. Drugs Interfering with Chromogenic Assays

### 2.1. Hydroxocobalamin

Hydroxocobalamin, given intravenously at high doses, is primarily indicated for treating cyanide poisoning in patients rescued from house fires. It has also been described as an off-label treatment for vasoplegia in critically ill patients [5].

The absorption spectrum of this vitamin B_12_ precursor is characterized by two peaks in ultraviolet light at 274 nm and 351 nm, two smaller confluent broad peaks in visible light at 500 and 526 nm, and no absorption above 600 nm. This spectral profile gives the drug a deep red color and is anticipated to interfere with chromogenic assays using detection wavelengths between 300 to 600 nm [6]. A single dose of hydroxocobalamin at the recommended level for the treatment of cyanide poisoning is enough to confer red plasmatic discoloration [6]. The drug remains in the body for weeks, as suggested by the persistence of urine discoloration, but its interfering potential on chromogenic assays performed on plasma seems to be shorter, 48 h or 72 h maximum in one study [7,8]. The impact of hydroxocobalamin on colorimetric assays depends on the wavelength used by the test and the analyte concentration. The closer the wavelength of the reaction is to the peak of absorption of hydroxocobalamin, the higher the interference will be. Conversely, increasing analyte concentration decreases the impact of the interferent on the final result [9]. A correlation between increasing concentrations of hydroxocobalamin and a higher degree of interferences has been demonstrated for many routine laboratory assays, including chemistry, co-oximetry, hematology, and coagulation instruments [10].

Plasma containing hydroxocobalamin mimics a hemolyzed sample, so the possibility of using the hemolysis index provided by some analyzers to detect and quantify the degree of interference has been evaluated [9]. The hemolysis index appears efficient in detecting the potentially interfering drug and estimating its concentration [9,10]. A limitation of using the hemolysis index is that it can also be increased by free hemoglobin by definition. Samples should be carefully evaluated for hemolysis, as it affects some chromogenic coagulation assays only by spectral interference (e.g., anti-Xa), while it may combine this mechanism with a spurious coagulation activation for PT and aPTT measured by optical or mechanical clot-based detection, although this effect is much less pronounced for mechanical detection [11].

Among routine coagulation testing, spectral interferences have been described for anti-Xa assays as well as PT and aPTT measured with an optical method [12]. Dang et al. investigated the interfering potential of hydroxocobalamin at therapeutic concentrations on several routine coagulation assays. The chromogenic Anti-Xa assay was affected by the drug, with an overall underestimation of 30% for baseline anti-Xa values close to 0.15 IU/mL [10]. PT and aPTT values were obtained using chronometric clot-based methods in this study. Surprisingly, both parameters were impacted by the presence of hydroxocobalamin in plasma, resulting in an overestimation of 45% for the former and 32% for the latter. These results suggest another mechanism in which this drug interferes with PT and aPTT, either by affecting hemostasis in vivo or by an unknown interference with these two chronometric assays. Anti-Xa assays measure the functional level of unfractionated heparin (UFH) in plasma through its anti-Xa activity. A test plasma sample from a patient on UFH is incubated with Factor Xa (FXa) in excess. The Xa is inactivated by the AT-Heparin complex, and residual Xa is assayed using a specific chromogenic substrate. The degree of coloration is proportional to the residual FXa concentration and then inversely proportional to anti-Xa activity. The wavelength used in the assay is usually below 600 nm, falling in the range of absorption of hydroxocobalamin, and is then susceptible to being influenced by the presence of hydroxocobalamin. Cagle et al. reported a significant interference between hydroxocobalamin and a chromogenic anti-Xa assay in a patient on mechanical circulatory support, in which anti-Xa was undetectable despite the use of UFH at the time of sampling [12]. Despite the limitations of this case report, the most likely explanation for the undetectability of anti-Xa for nine days is an optical interference caused by hydroxocobalamin, given that this duration correlates with the visibly red color of the patient’s chest tube and urine outputs.

### 2.2. Intravenous Lipid Emulsion and Propofol

Intravenous lipid emulsions (ILE) are part of the therapeutic arsenal in the ICU. They are mostly used for parenteral nutrition, which provides essential fatty acids and calories to patients who are unable to feed enterally, and for the administration of poorly water-soluble drugs where lipid emulsion acts as a diluent. In some institutions, ILE is also used as an antidote for lipophilic drug toxicity by trapping them in a lipid sink and reducing their bioavailability and toxicity [13]. Initially limited to local anesthetics such as bupivacaine, the use of ILE has expanded to other drugs with life-threatening toxicity [14]. In their large retrospective study on the frequency and causes of lipemia interferences on chemistry laboratory tests, Mainali et al. found that more than half of samples with a very high lipemic index were linked to lipid-containing intravenous infusions. Fat emulsions for parenteral nutrition account for most of the cases, followed by propofol [15].

Propofol is a short-acting anesthetic used in the ICU to sedate patients during mechanical ventilation. It is formulated as an oil-in-water emulsion and can interfere with chromogenic assays due to its milky white appearance. A series of five critically ill patients receiving continuous propofol infusion demonstrated major interferences on aPTT measured with a photo-optical method at 405 nm [16]. The interference caused erroneous shortened aPTTs with no reportable aPTT in all cases in comparison with reportable values before propofol for all patients. The time from the last propofol dose to a reportable aPTT ranged from 3.5 to 24 h, with a median of 7 h. PT and fibrinogen seemed less affected by the propofol. Sample ultracentrifugation and measurement at alternative wavelengths, such as 570 nm, could mitigate this interference in some cases. The authors suggest repeated aPTT measurement at least 4 h after stopping propofol to limit its impact. Negaards et al. carried out a retrospective observational cohort study of 38 critically ill patients receiving both propofol and heparin infusions, with their therapy monitored using an optical-based aPTT [17]. A total of 531 aPTT from these patients were monitored during the period of overlapping propofol and intravenous UFH. An interference was highlighted in 109 aPTT results obtained from 21 patients, with all affected patients having at least one aPTT requiring ultracentrifugation before reporting, and 12 aPTT from four patients being unreportable due to the spectral interference related to propofol. Like the previous study, ultracentrifugation removed the spectral interference for most samples but significantly delayed aPTT reporting, potentially impacting timely adjustments of heparin dosage. The analyzer used in this study offers a flag to identify samples with excessive lipemia that may interfere with coagulation testing. Surprisingly, it detected less than 5% of samples with propofol-induced optical aPTT interference.

An in vitro analysis confirms the sensitivity of coagulometers using optical detection to turbidity induced by ILE and the potential benefits of high-speed centrifugation of the sample before analysis or the use of an analyzer measuring clotting times based on mechanical detection [18]. Plasma from 19 healthy volunteers was spiked with intralipid 20% Fresenius Kabi (100 mL or 500 mL) to simulate different concentrations of an adult treatment and then tested with an optical-based coagulometer (CN-6000, Sysmex, Kobe, Japan) for international normalized ratio (INR), aPTT, and fibrinogen. This analyzer provides a lipemia index that defines whether a result is reportable or whether the sample is rejected. All results from samples containing a high concentration of intralipids were rejected for INR, aPTT, and fibrinogen, as well as half of the samples with a low concentration. The use of high-speed centrifugation allowed for reliable results for all low and high-concentration samples that were rejected on the first run. Clot-based assays provided reliable INR, aPTT, and fibrinogen results for all samples containing a low concentration of intralipids. All results for samples containing a high concentration of intralipid were reported, but with a slight overestimation for INR and aPTT ratio (0.98 ± 0.05 vs. 1.08 ± 0.12 and 1.06 ± 0.09 vs. 1.16 ± 0.09, respectively) and a slight underestimation for fibrinogen (2.69 ± 0.59 vs. 2.40 ± 0.46 g/L). These results suggest that optical interference is not the only mechanism by which lipid emulsions interact with routine coagulation assays. Notably, a high quantity of lipids correlates with less available water in the sample, and modification of the plasma/citrate ratio may explain the artefactual prolonged screening test clotting times [19].

Ultracentrifugation effectively reduces spectral interference due to lipemia but is not available in all laboratories and may be associated with the loss of high molecular weight proteins, such as fibrinogen, FVIII, and von Willebrand factor [20]. Gardiner et al. evaluated the possibility of using a high-speed centrifugation method to remove lipemia interference and allow testing with an optical-based coagulometer. Although ultracentrifugation eliminates more triglycerides and cholesterol than high-speed centrifugation (10,000× *g* for ten minutes), the latter was efficient enough to provide reliable results for aPTT, PT, and fibrinogen with analyzers using optical detection [20]. The usefulness of this high-speed centrifugation was confirmed on real-world samples the same year [21]. In this evaluation, all samples for which no result was provided on the first run were valid after the high-speed centrifugation procedure. Conversely, there seems to be no added value to apply this procedure when the instrument provides a result on the first run for PT, aPTT, and fibrinogen < 2 g/L. Note that the conclusion of this study may be limited to the analyzer evaluated in this study.

A retrospective evaluation of nine patients receiving ILE therapy for the lipophilic treatment of drug toxicity reported laboratory interferences for four of them, despite ultracentrifugation of the samples [13]. Turbidity prevented analysis of clinical chemistry tests for more than 16 h in two subjects and precluded organ donation in a third. The authors emphasized that serial ultracentrifugation did not overcome the spectral interference but did not propose an explanation. In addition to triglycerides, ILE solutions contain glycerol, which helps preserve physiological osmolarity but is also known to interfere with some triglyceride assays [22]. Centrifugation usually fails to remove glycerol, and samples from patients receiving ILE could benefit from glycerol blanking. Another case report published a year after presents a 43-year-old female with prolonged laboratory interference lasting up to 25 h following ILE therapy [23]. The authors hypothesized that the prolonged turbidity induced by high-dose ILE could be caused by a saturation of the reticuloendothelial system that metabolizes large lipid droplets, leading to decreased clearance of ILE.

Another controversial topic is the significant amount of vitamin K present in ILE, either for parenteral nutrition or as a drug delivery vehicle for propofol [24]. While some authors demonstrated a negligible effect on PT, others reported an impact strong enough to induce vitamin K antagonist (VKA) resistance. It led them to suggest closer monitoring and the replacement of ILE and propofol with an alternative agent when possible for patients on VKA treatment [25,26,27].

## 3. Drugs Interfering with Chronometric Assays

### 3.1. PEGylated Compounds

The process of conjugating polyethylene glycol to a drug molecule, known as PEGylation, is used in the pharmaceutical industry to prolong the circulating half-life of a drug by increasing its size and reducing renal clearance [28]. The FDA has approved a total of 38 PEGylated products so far, and at least as many are in active clinical trials [29]. Most of them are indicated for the treatment of cancers and blood diseases, disorders subject to numerous complications requiring admission to intensive care. The PEGylation process has been shown to prolong aPTT, and the emergence of extended half-life therapeutics based on this principle for the treatment of hemophilia A and B has greatly increased interest in this interference.

An analysis of the impact of PEG on aPTT assay was conducted on normal pool plasma spiked with clinically relevant concentrations of three PEGylated compounds, PEG alone, and compound alone, to determine whether there is any difference between six different aPTT reagents [30]. PEG-TNFalpha, PEG-albumin, PEG-hemoglobin, and PEG alone prolong only two aPTT assays, both using silica as an activator and cephalin as a source of phospholipids. The prolongation was more pronounced for STA-PTT A than for HemosIL aPTT, with a percentage change from baseline aPTT of up to 68% for the former and 12% for the latter. A few years later, Murphy et al. extensively investigated the effects of PEGylated compounds on aPTT and related assays to elucidate the mechanism of this interference [31]. Two PEGylated drugs with unrelated pharmacologic activity were used in the first part of the study to confirm that PEG prolongs aPTT in a dose-dependent manner for at least two weeks. Interestingly, PT and fibrinogen measured at the same time remained unchanged. Then, the use of a wide range of aPTT reagents with different sources and quantities of phospholipids, variable pH, and different types of activators supported the major contribution of silica activator to the aPTT prolongation induced by PEGylated compounds. The authors evaluated seven different aPTT reagents using silica as an activator, including five with micronized silica (STA-PTT A, PTT-LA, Triniclot aPTT HS, Triniclot aPTT S, and HemosIL aPTT Lyophilized silica) and two with colloidal silica (HemosIL SynthASil and HemosIL aPTT-SP). Prolongation of aPTT was observed with all of these reagents, except for one with colloidal silica activator (HemosIL SynthASil). The authors hypothesized that the polarity of the dispersion agent used for this reagent could modulate the adsorption of PEG onto the surface of colloid silica and mitigate the interference.

Over the past decade, new FVIII and FIX recombinant products with extended half-lives have been developed. These new drugs have improved patients’ quality of life by decreasing injection frequency and increasing trough levels. This innovation also interferes with one-stage clotting assays, which are based on aPTT, and may induce erroneous results that do not reflect the actual factor level. As of today, three PEGylated recombinant FVIII products (turoctocog alfa pegol, damactotog alfa pegol, and rurioctocog alfa pegol) and one PEGylated recombinant FIX product (nonacog beta pegol) are available on the market. FVIII levels are underestimated by most of the one-stage clotting assays using silica-based activators in patients receiving turoctocog alfa pegol or damactotog alfa pegol [32,33]. For turoctocog alfa pegol, an additional mechanism may be the decelerated activation of the drug by thrombin in the presence of silica [34]. The available data are inconsistent with rurioctocog alfa pegol. Surprisingly, a large overestimation of FIX levels has been reported, with most assays using silica as activator with nonacog beta pegol, and the main hypothesis being that the PEG group accelerates the conversion of the product into FIXa [32,33,35].

### 3.2. Emicizumab

Emicizumab is a recombinant humanized bispecific antibody that binds both activated FIXa and FX and mimics activated FVIII cofactor function. It is widely used as prophylaxis of bleeding episodes in patients with hemophilia A of all ages, with or without inhibitors. This drug has improved patient management by eliminating the need for regular biological monitoring. However, monitoring is still necessary in certain situations, like surgery or bleeding. Understanding how emicizumab affects coagulation assays is necessary to effectively guide treatment.

The interaction of emicizumab with human FIXa and FX is responsible for interferences in all tests involving these two human factors. Emicizumab shortens aPTT and falsely increases FVIII levels measured by one-stage aPTT-based assays and chromogenic assays using human-derived factor components [36]. Interestingly, this interference can be used to measure emicizumab levels after replacing the factor VIII plasma calibrator with the emicizumab calibrator [37].

Emicizumab interferes with all aPTT-based assays. Bowyer et al. reported significant aPTT shortening in plasma artificially spiked plasma to all 13 aPTT reagents tested [38]. In addition, aPTTs normalized after the first dose of emicizumab in samples from hemophilia A patients with inhibitors, with the seven aPTT reagents tested subsequently. Shortening of aPTT is dose-dependent, and values below the normal reference range can be observed even at low concentration, given the high sensitivity of aPTT reagents to emicizumab [39]. Nevertheless, normalization of aPTT should not be equated with the restoration of normal hemostasis, as pharmacokinetic studies show thrombin generation similar to a moderate or mild phenotype at therapeutic plasma concentrations [40]. Emicizumab may interfere with this screening assay for several months after the last dose due to its extended half-life. However, its effect can be completely neutralized by pre-treatment of the sample with a specific antibody [41].

Emicizumab has no effect on thrombin time (TT) or the Clauss fibrinogen assay, while increasing concentrations correlate with a slight increase in INR that is unlikely to be of clinical relevance [39]. Anti-Xa assays are unaffected by this drug and should therefore be preferred to aPTT for monitoring heparin therapy in critically ill patients. Due to the sensitivity of aPTT to emicizumab, all chronometric assays using an intrinsic pathway trigger are affected by it. Adamkewicz et al. found activity levels of FIX, FXI, and FXII to be dramatically overestimated, with values being already twice as high as controls for subtherapeutic concentration of emicizumab. FVIII-depleted plasma containing an emicizumab concentration of 25 µg/mL is associated with FVIII activity of 250% [42]. As explained previously, this result does not reflect the effect of emicizumab in vivo and should rather be considered as falsely elevated FVIII activity levels. Therefore, one-stage FVIII assays are not recommended for monitoring patients treated with emicizumab unless calibrated with emicizumab-specific calibrators. The ability to measure FVIII activity by chromogenic assays in such patients depends on whether human or animal reagents are used. Assays using human recombinant reagents show a concentration-dependent increase in FVIII activity due to the interaction of emicizumab with human FIXa and FX. In contrast, chromogenic methods using animal reagents or combined human/animal reagents are unaffected by emicizumab and are suitable for patients receiving FVIII concentrate replacement therapy in combination with emicizumab to measure residual factor VIII level and can notably be used to titrate factor VIII-specific inhibitors with the Bethesda method [43].

### 3.3. Recombinant Activated Factor VII (rFVIIa)

Hemorrhage is a major concern in critically ill patients, and recombinant activated factor VII (rFVIIa) is frequently used in this setting to control bleeding. In addition to the treatment of bleeding in patients with hemophilia and high-titer inhibitors, rFVIIa is routinely used in cases of traumatic brain injury, cirrhosis, and nontraumatic intracranial hemorrhage [44]. Administration of rFVIIa at a therapeutic concentration increases total circulating FVIIa approximately 100-fold, leading to a FVII:FVIIa ratio in peripheral blood of approximately 1:1. It initiates thrombin generation at the site of injury by two different mechanisms, through the tissue factor pathway after its release from damaged endothelium and through a TF-independent pathway by directly activating FIX [45]. These two modes of action explain why both PT and aPTT can be impacted by rFVIIa. Prediction of clinical effectiveness based on the shortening of these screening assays is unclear, likely due to the endpoint of these tests and the variability in laboratory response to this product [46,47].

rFVIIa shortens PT in a dose-dependent manner and follows the same course as the elevation of FVII activity [48]. Modification of PT occurs within minutes following administration of rFVIIa. Several authors highlighted an improved PT for all patients receiving rFVIIa without this being linked to the clinical efficacy of this bypass agent therapy. However, PT could be shorter for patients who respond in terms of hemostasis, and PT normalization appears to be a better indicator of treatment efficacy [49]. Patients in whom hemostasis was restored by rFVIIa without PT reduction are exceptional, so the absence of shortening may be useful in identifying non-responders. Other tricky criteria to consider are the temperature and pH dependence of rFVIIa. Acidosis and hypothermia could decrease its efficacy, and the controlled temperature and pH of PT assays may not reflect the patient’s actual condition.

Multivariate analysis of 47 cardiac surgery patients receiving rFVIIa for uncontrolled hemorrhage shows an association of aPTT and PT with rFVIIa-mediated hemostasis, with respective odds ratios of 0.18 [95% CI 0.05–0.72] and 0.10 [95% CI 0.01–0.98] [50]. In addition, aPTT normalization correlated best with rFVIIa efficacy despite being unrelated to total chest tube output in the two hours following administration. In contrast, a retrospective analysis of 293 cardiac surgery patients failed to link aPTT with rFVIIa efficacy and reported only an association with mortality in univariate analysis [51]. aPTT clot wave form analysis may improve the prediction of rFVIIa efficacy in patients with severe hemophilia A, as suggested in an in vitro evaluation [52].

FVII is usually increased well above normal values with a therapeutic dose of rFVIIa because it measures total plasma FVII activity [53]. A study including six cirrhotic patients undergoing orthotopic liver transplantation reported a dramatic increase in FVII activity following rFVIIa administration, with levels ten times higher than controls [54]. Factors II, VIII, and X activities were also overestimated in this study, but to a lesser extent. One manufacturer offers an assay to specifically measure FVIIa (Staclot VIIa-rTF), which could be suitable for estimating plasmatic rFVIIa concentration [55].

### 3.4. Antibiotics

Antibiotics occasionally interfere with routine coagulation testing. Here, we present the potential interference of three antibiotics with an activator or phospholipid-dependent mechanism, and a fourth that interacts with hemostasis in vivo and may expose patients to bleeding complications.

Daptomycin is a lipoglycopeptide antibiotic that exerts its antibacterial activity by integrating into the phospholipid membrane of gram-positive bacteria in a calcium-dependent manner. Several authors have reported a prolongation of PT linked to daptomycin, and the absence of bleeding in patients receiving this treatment suggests analytical interference [56]. In their in vitro evaluation, Webster et al. evaluated the impact of daptomycin on PT using 30 different thromboplastin reagent kits and found a dose-dependent effect on two recombinant thromboplastin reagents [57]. Reagents containing recombinant tissue factor are lipidized with phospholipids, and daptomycin is thought to interfere with the clotting process by integrating itself into these phospholipids. The concentration of phosphatidylglycerol in the lipid mixture appears to be the main contributor of the daptomycin-PT interference in spiked samples from patients with normal and prolonged PT [58]. A subsequent study focusing on one reagent with recombinant thromboplastin showed an INR falsely increased by 43% between expected peak and trough concentrations of daptomycin [59]. Their Monte Carlo simulation predicted that daptomycin concentrations capable of interfering with PT were low two hours after infusion, even with a high dose or in patients with creatinine clearance < 30 mL/min. The extent of the daptomycin-induced interference was reduced by co-administration of liposomal amphotericin B or an empty cationic liposome, a possible vector for therapy, both containing phosphatidylglycerol. A prospective study including 35 patients confirmed the dose-dependent artificial prolongation of PT due to daptomycin, which could be overestimated by up to 20% for a reagent containing recombinant human tissue factor [56]. In contrast to previous studies, a reagent containing rabbit brain was also affected by daptomycin without a satisfactory explanation being found. TT and aPTT seemed unaffected by this antibiotic [57].

Another lipoglycopeptide antibiotic known as telavancin may have the ability to bind to artificial phospholipids found in routine coagulation assays [60,61,62]. A case report showing an absence of bleeding in a patient having a telavancin-induced overestimation of INR reinforces the hypothesis of an interference [62]. Plasma levels of telavancin range from approximately 10 µg/mL at trough to 100–150 µg/mL at peak. Normal pool plasmas spiked with increasing concentrations of the antibiotic were used to assess its interference potential on different PT and aPTT reagents [60]. All reagents exhibited a dose-dependent effect of telavancin, but to a different extent depending on the case. For INR testing, one reagent containing recombinant thromboplastin had a dramatic overestimation between 10% at trough levels and up to 500% at peak levels. INR difference for the two other reagents was negligible at trough and did not exceed 35% at expected peak levels. The authors suggested a greater affinity for telavancin to a particular phospholipid type, present in variable quantities in the different PT reagents, to explain these differences. The results with aPTT reagents are more homogeneous, with a minimal effect at trough and a false increase of up to 100% at peak. Similar conclusions were drawn in a study involving 16 PT reagents and seven aPTT reagents, and another involving point-of-care coagulation instruments [61,63]. Altogether, these finding supports the need to draw blood before the next dose of telavancin for aPTT and PT testing or to switch to a chromogenic anti-Xa assay for UFH monitoring.

Aminoglycosides are widely used in patients with severe infections and can cause artifactual prolongation of aPTT due to interference with ellagic acid-based reagents [64,65]. Kaneko et al. reported a case of a 74-year-old man with chronic kidney disease and bacteremia having prolonged aPTT but normal PT after amikacin treatment, without bleeding symptoms [64]. A subsequent in vitro study confirmed that amikacin and gentamicin prolong aPTT in a dose-dependent manner with ellagic acid-based reagents, but not with silica as activator. The main hypothesis for this interference is copper ion chelation. A second case report goes in the same direction and provides some details on the extent of overestimation in the case of tobramycin administration [65]. In comparison with an aPTT unaffected by this antibiotic, the reagent using ellagic acid as an activator demonstrated a falsely prolonged result almost four times higher than the control at peak and 40% at trough.

Tigecycline is a broad-spectrum antibiotic mainly used against drug-resistant bacteria. Coagulopathy is a known side effect and can be accompanied by severe bleeding. Several authors demonstrated a dose-dependent prolongation of PT and aPTT, and a decrease in fibrinogen levels [66,67,68,69]. Treatment duration is an important risk factor for tigecycline-induced coagulopathies, and tigecycline discontinuation allows routine coagulation parameters to recover within 10 days in most cases [68,70]. A nomogram has been published recently to identify patients receiving tigecycline at high risk of developing a coagulopathy [70]. Intake of tigecycline for more than seven days, along with initial PT and fibrinogen levels, are among the independent predicting variables included in the score. A retrospective study pursued in 2024 with 920 patients from a single center found an incidence of tigecycline-induced coagulopathy of 24%. Alterations of PT, aPTT, and fibrinogen levels occurred on days 5 to 7 after administration, and the changes were more pronounced as the dose was high [66]. The most dramatic change was observed with fibrinogen levels, with values dropping by up to 50% in comparison with initial levels. Several mechanisms have been proposed to explain the effect of tigecycline on hemostasis [71]. It may inhibit interleukin-6 synthesis or modify microRNA-122 levels, both involved in sepsis-induced coagulopathies. The drug could also interfere with vitamin K metabolism, either by disrupting gut microbiota or directly inhibiting the activity of vitamin K. This latter hypothesis is the most likely, as the use of vitamin K_1_ in 72 patients with tigecycline-induced coagulopathy resulted in an improvement of all routine coagulation parameters [66]. Compared with pre-treatment values, aPTT was reduced by approximately 20%, PT by 12%, and fibrinogen levels by 35%.

### 3.5. Sugammadex

Sugammadex is modified gamma-cyclodextrin administered in the postoperative period to reverse the effects of certain muscle relaxants, such as rocuronium or vecuronium. It is used in the ICU for short bedside procedures requiring neuromuscular blockade followed by reversal, with the most common settings being bronchoscopy, percutaneous dilatative tracheostomy, and percutaneous endoscopic gastrostomy [72].

A randomized controlled trial carried out by De Kam et al. on eight healthy subjects reported transient prolongation of aPTT and PT up to 22%, with sugammadex 20 mg/kg that returned to baseline values within 30 min post-administration [73]. Additional in vitro assays confirmed this finding and showed a concentration-dependent prolongation of aPTT and PT, reaching, respectively, 29% and 19% at the maximum therapeutic concentration of the drug. The same conclusion is reached for surgical patients, with a slight prolongation of the two assays likely due to the lower dose of sugammadex (i.e., 4 mg/kg) [74]. aPTT increased by 5.5% and PT by 3.0% from baseline 10 minutes after administration of sugammadex, and values returned to normal within 60 min. Other blood loss indicators (such as transfusion rates, 24 h drain volume, anemia, and drop in hemoglobin) were similar in patients receiving sugammadex and standard care, suggesting a lack of additional bleeding risk in surgical patients receiving this drug. Other authors support this conclusion despite the moderate and transient prolongation of aPTT and PT, as well as its impact on thromboelastographic parameters [75,76,77].

**Figure 1:**
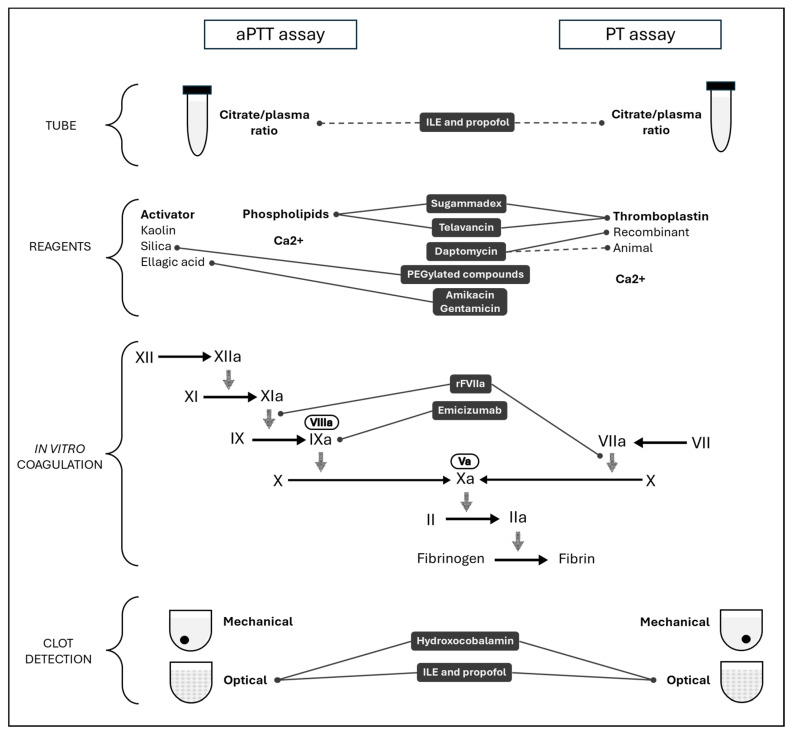
Mechanism of interferences related to drugs used in intensive care on activated partial thromboplastin time (aPTT) and prothrombin time (PT).

The effect of sugammadex on hemostasis may be attributed to an in vitro artifact due to a phospholipid binding [78]. In addition to a dose-dependent effect on aPTT, Dirkmann et al. showed a prolongation of the dRVVT assay with low phospholipid concentration that was significantly mitigated by the additional phospholipids in a second dRVVT assay. This mechanism could explain the effect of sugammadex on phospholipid-dependent assays. Table 1 lists drugs used in intensive care and potentially interfering with routine test results described in this review. In addition, Figure 1 illustrates the specific impact of each drug on aPTT and PT assays, and whether it affects the citrate/plasma ratio, reagents, in vitro coagulation, or clot detection.

## 4. Drugs Affecting the Hemostasis System

### 4.1. Bridging Anticoagulation

Long-term antithrombotic therapy is indicated in a myriad of clinical conditions, including atrial fibrillation, venous thromboembolism, mechanical heart valves, coronary artery disease, and peripheral arterial diseases. Prevalence of these conditions is increasing worldwide, notably due to population aging. The number of patients on long-term treatment who need surgery or invasive procedures is increasing similarly. It is estimated that 15 to 20% of these patients will require these procedures annually, and 10 to 15% of patients with coronary stents will require surgery within two years of implantation [79]. “Anticoagulant bridging” refers to the administration of an alternative anticoagulant with a short half-life, usually heparin (most often low-molecular-weight heparin (LMWH), more rarely UFH), to ensure safe, efficient, and controlled anticoagulation in the acute setting. The American College of Chest Pathologists guidelines on perioperative management of antithrombotic therapy recommend against the use of heparin bridging in patients with atrial fibrillation [79]. Similarly, no benefit of bridging in patients with mechanical heart valves has been demonstrated in the PERIOP2 study or in patients with cancer-associated thrombosis [80,81]. However, according to the latest European Society of Cardiology guideline, bridging can be considered in patients with a mechanical prosthetic heart valve, patients with significant mitral stenosis, patients with an acute thrombotic event within the previous four weeks, and also patients with ‘high acute thromboembolic risk’ (patients with a CHA2DS2-VASc score > 3 for women or >2 for men) [82]. Recently, authors proposed to restrict bridging in the last category, i.e., patients with high thromboembolic risk, to patients with a very high thrombotic risk (e.g., CHA2DS2-VASc ≥ 7) [83].

In patients with anticoagulant bridging, assessment of the level of anticoagulation may be desired. Long-term treatment may include VKAs or direct oral anticoagulants (DOACs) specifically inhibiting either FXa (apixaban, edoxaban, rivaroxaban) or FIIa, aka thrombin (dabigatran). VKAs are routinely monitored with the INR, which is derived from the PT. Almost all PT reagents contain a heparin neutralizer to allow for warfarin monitoring during concurrent heparin therapy [84]. In contrast, the aPTT cannot be used as long as the result of VKA administration, i.e., reduced activity of vitamin K-dependent coagulation factors, i.e., factors II, IX, and X (Factor VII level does not impact aPTT), is present. The only way of monitoring heparin (UFH and LMWH) is the anti-Xa assay. In addition, potential advantages of anti-Xa level assay over aPTT for heparin monitoring have been identified. This includes fewer interferences (e.g., elevated C-reactive protein, lupus anticoagulant, high factor VIII, or fibrinogen levels), fewer monitoring tests, fewer dose changes, and a shorter time to obtain therapeutic anticoagulation and similar bleeding rates [85,86]. In particular, anti-Xa can be preferred to aPTT in ICU patients with an increased risk of bleeding [87].

DOACs have a short half-life, and algorithms are proposed to ensure DOAC flushes out before intervention or bridging when necessary. The timing of DOAC discontinuation to ensure a safe procedure depends on the nature of the DOAC (dabigatran vs. apixaban, edoxaban, or rivaroxaban), the renal function (creatinine clearance), and the risk of bleeding (low vs. high) [83]. Nevertheless, DOACs may accumulate in patients with comorbidities and comedications, and a complete elimination of the drug may not be achieved following the proposed algorithms [88]. Residual circulating DOACs may then interfere with routine assays, PT, aPTT, fibrinogen, and TT. The level of interference may depend on the DOAC and the reagent used [89]. Of note, a significant level of circulating DOAC cannot be excluded by a normal PT or aPTT. In contrast, TT is usually highly sensitive to very low levels of dabigatran, while direct FXa inhibitors have no impact on TT due to their mechanism of action [90]. As a consequence, routine assays cannot be used to assess a DOAC circulating level, nor to rule out the presence of a residual DOAC concentration. Finally, the fibrinogen assay is based on the cleavage of fibrinogen present in the test sample by exogenous thrombin contained in the reagent. The more thrombin the reagent contains, the less sensitive to dabigatran is the fibrinogen assay. The fibrinogen assay results in patients on dabigatran should then be interpreted with caution, especially if a reagent containing a relatively low concentration of thrombin is used [91]. DOAC levels are measured only in selected patients and in emergency situations. Direct FXa inhibitors are usually measured using an anti-Xa assay, with a dedicated test set-up and calibrators and controls. The endpoint of the assay is the measurement of the residual FXa using a specific chromogenic substrate. As direct FXa inhibitors exhibit a significantly higher anti-Xa activity compared to heparin, the specific test set-up includes a higher dilution of the plasma sample [89]. The interference of heparin in the direct FXa assay is then minimized, if not abolished. A modified anti-Xa assay, including a chaotropic buffer preventing the binding of heparin to antithrombin and inhibiting heparin anti-Xa activity, has been proposed as a direct FXa inhibitor. This assay allows the specific quantitation of direct Xa inhibitors in the case of heparin bridging [92]. On the contrary, one may want to evaluate the heparin anticoagulant activity in patients with a concomitant circulating direct FXa inhibitor. This could be achieved by measuring the heparin anti-IIa activity in case of bridging with UFH, as by definition UFH exhibits an anti-Xa/anti-IIa ratio of one. However, the lack of commercial anti-IIa assays is a limitation to this approach. In addition, evaluating the actual hemostatic status of a patient with different anticoagulants onboard is highly challenging, as no direct correspondence exists between the various anticoagulants.

Specialized assays, e.g., antithrombin, protein C, or protein S activity assays, or activated protein C resistance assay, can be based on a clotting or a chromogenic assay. As DOACs may prolong clotting time, they may interfere with such clotting assays. Chromogenic assays may rely on the measurement of residual Factor Xa or IIa, i.e., thrombin, using a specific chromogenic substrate. Direct FXa inhibitors can then interfere with assays measuring residual FXa as the endpoint, while dabigatran may interfere with assays using thrombin as the endpoint. In contrast, immunoassays are not affected by the presence of DOACs [93]. Table 2 summarizes the interpretation of routine test results in the case of bridging.

Argatroban is a parenteral direct thrombin inhibitor that is being used increasingly in intensive care medicine for patients with heparin-induced thrombocytopenia or, more recently, vaccine-induced immune thrombotic thrombocytopenia. As with dabigatran, argatroban can prolong aPTT and can affect Clauss fibrinogen assays and chromogenic assays based on the measurement of FIIa [94,95].

Novel anticoagulant drugs targeting the contact phase factors, namely FXI, FXIa, FXII, and FXIIa, are currently under development. They are characterized by a variety of chemical structures and mechanisms of action, various routes of administration, and very diverse half-lives. No specific assay is currently available, but aPTT can be prolonged depending on the reagent, the nature of the drug, and its concentration [96,97]. It is still unknown if bridging may be of interest from a clinical standpoint in some patient populations, but one can anticipate that, if needed, assessment of the patient’s anticoagulant status may also be challenging.

### 4.2. N-Acetylcysteine

N-acetylcysteine (NAC) is an effective antidote for acetaminophen intoxication if administered within 10 h of ingestion. It acts as a substitute or precursor to glutathione, an antioxidant that neutralizes the toxic metabolite N-acetyl-p-benzoquinone imine (NAPQI). NAC may have other indications in the ICU, such as other causes of hepatotoxicity, sepsis, acute respiratory distress syndrome, and the prevention of radiographic contrast-induced nephropathy [98]. This drug decreases the activity of vitamin K-dependent coagulation factors, possibly by reducing disulfide bridges in glycoproteins. It inhibits vitamin K epoxide reductase (VKOR) activity at therapeutic concentrations used for acetaminophen intoxication treatment.

Prolongation of PT can occur with acute acetaminophen overdoses without any sign of hepatotoxicity, due to NAPQI inhibiting vitamin K-dependent γ-carboxylase and VKOR enzymes [99]. This prolongation is time- and dose-dependent and correlates with low levels of functional FVII. Two-thirds of patients with extrapolated 4 h acetaminophen concentrations above 150 mg/L have an abnormal INR at some point, while this proportion falls to one-third for patients with concentrations below 150 mg/L [100]. Several studies show that intravenous NAC itself may prolong PT. In their investigation of the effect of acetylcysteine on PT in patients with uncomplicated acetaminophen poisoning, Schmidt et al. found that PT increased in all patients after NAC infusion, unrelated to acetaminophen ingestion [101]. Evolution of PT in 18 patients with benign paracetamol overdose receiving NAC shows a fall in TP for all patients 14 h after administration, with values below normal for a third of them, and all PT returning to baseline after stopping NAC infusion [102]. In vitro studies support the correlation between increasing NAC concentrations and PT prolongation, whereas this drug appears to have no effect on aPTT [103,104]. As expected, NAC significantly reduces the activity of factors II, VII, IX, and X, with values being reduced by up to 63% compared to baseline in plasma from healthy subjects spiked with therapeutic concentrations of NAC.

PT is a sensitive prognostic marker for acute liver failure and is part of the King’s College criteria for determining which patients should undergo liver transplantation [105]. In patients with uncomplicated acetaminophen intoxication who are receiving NAC infusion, a decrease in the PT index might be misinterpreted as a sign of liver failure [101,102]. Physicians should be cautious when interpreting PT values that are near critical, relevant clinical thresholds.

### 4.3. Corticosteroids

Corticosteroids are beneficial for a wide range of critically ill patients due to their anti-inflammatory properties. In this setting, their effects on hemostasis are difficult to assess because several coagulation parameters are considered acute-phase reactants.

In their systematic review, Van Zanne et al. assessed the effect of corticosteroids on coagulation parameters by differentiating studies including patients without other potentially influencing factors from those with increased inflammatory activity [106]. A 5-day course of dexamethasone in healthy volunteers was associated with a slight increase in fibrinogen, FVII, FVIII, and FXI, with changes ranging from +6% to +27%, while D-dimer and VWF results remained unchanged by the treatment [107]. Two recent investigations involving higher doses of corticosteroids for a longer period confirmed the lack of effects on D-dimers, but reached different conclusions for fibrinogen, which decreased by 15% in one study, and for VWF, which increased by 20–30% in both studies [108,109]. A possible explanation for these discrepancies could be the lower dose and treatment duration in the first study, which were not sufficient to increase VWF synthesis and release from vasculature endothelial cells [109]. Fibrinogen and VWF, both considered markers of inflammation, were reduced after the use of corticosteroids in patients with an underlying inflammatory disorder in several studies [106]. This could be better explained by the anti-inflammatory effect of corticosteroids on acute-phase reactants rather than a direct effect of the drug itself. Corticosteroids also alter the hemostasis balance by inhibiting fibrinolytic activity, due to increased levels of PAI-1, which could contribute to the prothrombotic state in patients receiving this treatment [106,108]. PT and aPTT seem unaffected by glucocorticoids in healthy volunteers and critically ill patients with systematic inflammation [106,109].

### 4.4. Serotonin Reuptake Inhibitors and Tramadol

Selective serotonin reuptake inhibitors (SSRIs) (e.g., sertraline, citalopram, escitalopram, fluoxetine, and paroxetine) are widely used for the treatment of depression, obsessive-compulsive disorder, and other psychiatric conditions. Tramadol is a weak μ-opioid receptor agonist which acts as an SSRI, and is used as an analgesic for both acute and chronic pain, including postoperative persistent pain, cancer-related pain, and lower back pain. SSRIs are associated with an increased bleeding risk as they primarily interfere by decreasing platelet aggregation by 50% or more. They can also induce or worsen thrombocytopenia, contributing to a more pronounced bleeding phenotype [110]. A study published in 2011 suggested that SSRIs could also interfere with coagulation and fibrinolysis, contributing to excessive bleeding [111]. This effect could translate into a slight reduction in fibrinogen levels and a slight aPTT prolongation.

## 5. Conclusions

Coagulation pathways are intrinsically complex. Assays to assess the hemostatic balance and assess both the bleeding and the thrombotic risks are then essential for adequate, safe, and cost-effective patient management. However, pitfalls may hamper the validity of diagnosis. Difficulties may arise from interferences induced by various endogenous or exogenous substances, including medications. Drugs are at the frontline for curing patients, especially in intensive care settings, where pathophysiological manifestations may make patient management even more challenging. Detecting possible interferences of various natures in medical laboratory assays, specifically in coagulation assays, and awareness of how these medications may per se interfere with coagulation pathways in vivo is crucial to manage patients in critical care units.

## Figures and Tables

**Table 1:** Potential interferences of drugs used in intensive care on routine test results.

Drug	Type of Interference	PT/INR	aPTT	Fibrinogen #	Anti-Xa
Hydroxocobalamin *	Chromogenic (similar to hemolysis)	±	±	±	↘
ILE and propofol *	Chromogenic (lipemia)	±	±	±	↘
PEGylated compounds	Chronometric (silica-based reagents)	ns	↗	ns	ns
Emicizumab	Chronometric (mimics FVIIIa cofactor function)	ns	↘	ns	ns
rFVIIa	Chronometric (TF dependent and independent activation)	↘	↘	ns	ns
Daptomycin **	Chronometric (phospholipid-dependent)	±	ns	ns	ns
Telavancin ***	Chronometric (phospholipid-dependent)	±	↗	ns	ns
Gentamicin and amikacin	Chronometric (ellagic acid-based reagents)	ns	↗	ns	ns
Sugammadex	Chronometric (phospholipid-dependent)	↗	↗	ns	ns

* Prolongation of PT/INR and aPTT and reduction in fibrinogen levels measured by optical clot-based detection. Unclear impact on assays using chronometric detection; ** PT prolongation depending on the reagent used; *** aPTT prolongation homogeneous among the different reagents tested, while PT prolongation is more pronounced with reagents containing recombinant thromboplastin; # Clauss method; ns indicates clinically not significant changes, ↗ indicates an overestimation, ↘ indicates an underestimation, ± indicates possible interference depending on the reagent or analyzer used.

**Table 2:** Interpretation of routine test results in the case of bridging.

Long-Term Drug	Bridging	What Does Assay Result Reflect?			
		PT (INR for VKA) *	aPTT **	Fibrinogen ***#	Anti-Xa ****
VKA	UFH	VKA ± UFH	VKA + UFH	Fib	UFH
	LMWH	VKA ± LMWH	VKA ± LMWH	Fib	LMWH
DXai	UFH	DXai ± UFH	DXai ± UFH	Fib	DXai ± UFH
	LMWH	DXai ± LMWH	DXai ± LMWH	Fib	DXai ± LMWH
Dabigatran	UFH	Dabi ± UFH	Dabi ± UFH	Fib ± dabi	UFH
	LMWH	Dabi ± LMWH	Dabi ± LMWH	Fib ± dabi	LMWH

* Prolongation of PT (INR) in the presence of UFH or LMWH depends on the heparin neutralizer present in the PT reagent and on the level of heparin in the plasma sample, as well as on the PT reagent used and DXai or dabigatran concentration present in the plasma sample. ** Prolongation of aPTT in the presence of LMWH depends on the nature and circulating level of LMWH (the lower the LMWH preparation anti-Xa/anti-IIa ratio, and the higher the circulating LMWH level, the greater the aPTT prolongation); interference of the DXai or dabigatran also depends on the aPTT reagent and the nature and concentration of the DXai or circulating concentration of dabigatran. *** The impact of dabigatran on the fibrinogen assay depends on the thrombin concentration in the fibrinogen reagent for the Clauss method and on the circulating dabigatran concentration; dabigatran will result in fibrinogen concentration underestimation in case of interference. DXai may also interfere with fibrinogen measured with the PT-derived method, depending on the PT reagent and nature and concentration of the DXai. **** Anti-Xa will reflect the combination of both DXai and UFH or LMWH, except in the case of an anti-Xa assay specifically for DXai; **#** Clauss method.

## Data Availability

Not applicable.

## Abbreviations

The following abbreviations are used in this manuscript:

aPTT	Activated partial thromboplastin time
DOAC	Direct oral anticoagulant
DXai	Direct Factor Xa inhibitor
Fx	Factor x (e.g., FVIII for factor VIII)
HIT	Heparin-induced thrombocytopenia
ICU	Intensive care unit
ILE	Intravenous lipid emulsion
INR	International normalized ratio
LMWH	Low-molecular-weight heparin
NAC	N-acetylcystein
NAPQI	N-accetyl-para-benzoquinoneimine
PT	Prothrombin time
rFVIIa	Recombinant activated factor VII
TT	Thrombin time
UFH	Unfractionated heparin
VKA	Vitamin K antagonist
VKOR	Vitamin K epoxide reductase
